# CSF total tau/α-synuclein ratio improved the diagnostic performance for Alzheimer’s disease as an indicator of tau phosphorylation

**DOI:** 10.1186/s13195-020-00648-9

**Published:** 2020-07-13

**Authors:** Kyu Hwan Shim, Min Ju Kang, Jee Won Suh, Jung-Min Pyun, Nayoung Ryoo, Young Ho Park, Young Chul Youn, Jae-Won Jang, Jee Hyang Jeong, Kyung Won Park, Seong Hye Choi, Kyoungho Suk, Ho-Won Lee, Pan-Woo Ko, Chan-Nyoung Lee, Tae-Sung Lim, Seong Soo A. An, SangYun Kim, for the Alzheimer’s Disease All Markers (ADAM) Research group

**Affiliations:** 1 Department of Neurology, Veterans Medical Research Institute, Veterans Health Service Medical Center, Seoul, Republic of Korea; 2 Department of Neurology, Seoul National University Bundang Hospital and Seoul National University College of Medicine, Seongnam-si, Gyeonggi-do Republic of Korea; 3 Department of Neurology, Chung-Ang University Hospital, Seoul, Republic of Korea; 4 Department of Neurology, Kangwon National University Hospital, Kangwon National University School of Medicine, Chouncheon, South Korea; 5 Department of Neurology, Ewha Womans University Mokdong HospitalEwha Womans University, Seoul, Republic of Korea; 6 Department of Neurology, Dong-A University College of Medicine and Institute of Convergence Bio-Health, Busan, Republic of Korea; 7 Department of Neurology, Inha University School of Medicine, Incheon, Republic of Korea; 8 Department of Pharmacology, Kyungpook National University School of Medicine, Daegu, Republic of Korea; 9 Department of Neurology, Kyungpook National University School of Medicine, Daegu, Republic of Korea; 10 Department of Neurology, Korea University Medicine, Seoul, Republic of Korea; 11 Department of Neurology, Ajou University School of Medicine, Suwon, Republic of Korea; 12 Department of Bionano Technology, Gachon University, Seongnam-si, Gyeonggi-do Republic of Korea

**Keywords:** Alzheimer’s disease, Cerebrospinal fluid, Tau, α-Synuclein, Biomarker

## Abstract

**Background:**

Recently, several studies suggested potential involvements of α-synuclein in Alzheimer’s disease (AD) pathophysiology. Higher concentrations of α-synuclein were reported in cerebrospinal fluid (CSF) of AD patients with a positive correlation towards CSF tau, indicating its possible role in AD. We analyzed the CSF biomarkers to verify whether α-synuclein could be an additional supported biomarker in AD diagnosis.

**Methods:**

In this cross-sectional study, CSF samples of 71 early-onset AD, 34 late-onset AD, 11 mild cognitive impairment, 17 subjective cognitive decline, 45 Parkinson’s disease, and 32 healthy control (HC) were collected. CSF amyloid-β1-42 (A), total tau (N), and phosphorylated tau181 (T) were measured by commercial ELISA kits, and in-house ELISA kit was developed to quantify α-synuclein. The cognitive assessments and amyloid-PET imaging were also performed.

**Results:**

CSF α-synuclein manifested a tendency to increase in AD and to decreased in Parkinson’s disease compared to HC. The equilibrium states of total tau and α-synuclein concentrations were changed significantly in AD, and the ratio of total tau/α-synuclein (N/αS) was dramatically increased in AD than HC. Remarkably, N/αS revealed a strong positive correlation with tau phosphorylation rate. Also, the combination of N/αS with amyloid-β1-42/phosphorylated tau181 ratio had the best diagnosis performance (AUC = 0.956, sensitivity = 96%, specificity = 87%). In concordance analysis, N/αS showed the higher diagnostic agreement with amyloid-β1-42 and amyloid-PET. Analysis of biomarker profiling with N/αS had distinctive characteristics and clustering of each group. Especially, among the group of suspected non-Alzheimer’s disease pathophysiology, all A−T+N+ patients with N/αS+ were reintegrated into AD.

**Conclusions:**

The high correlation of α-synuclein with tau and the elevated N/αS in AD supported the involvement of α-synuclein in AD pathophysiology. Importantly, N/αS improved the diagnostic performance, confirming the needs of incorporating α-synuclein as a biomarker for neurodegenerative disorders. The incorporation of a biomarker group [N/αS] could contribute to provide better understanding and diagnosis of neurodegenerative disorders.

## Background

The prevalence of various neurodegenerative disorders, including Alzheimer’s disease (AD) and Parkinson’s disease (PD), has increased dramatically worldwide, as the elderly population grew [1–3]. AD was pathologically characterized by accumulations of amyloid-β extracellular aggregates and intraneuronal hyperphosphorylated tau protein in the nervous system. However, since AD could be caused by complex mechanisms through the intersections with other proteins, alternative triggers in place of Aβ and tau have recently been explored.

Since α-synuclein (α-syn) was a major component of the Lewy body in PD and dementia with Lewy bodies, it became a representative biomarker for the related neurodegenerative disorders with parkinsonism [4, 5]. On the other hand, several reports suggested potential associations between α-syn and AD. Lewy bodies with α-syn were present in 40–50% of AD patients, whom revealed faster cognitive declines [6–9]. In animal models, α-syn overexpressing mice caused memory impairments similar to AD mouse models, and the accelerated cognitive declines were also observed in α-syn mutant mice [10–12].

Amyloid-β1-42 (Aβ_42_), total tau (T-tau), and phosphorylated tau181 (P-tau_181_) in cerebrospinal fluid (CSF) have been used primarily as biomarkers of AD. However, accurate diagnosis and appropriate treatments were difficult due to the co-presences with other neurodegenerative disorders, such as dementia with Lewy bodies, frontotemporal dementia, and PD [13–15]. The incorporation of new biomarkers, such as α-syn or TDP-43, should be demanded, as there were limitations to distinguish and to interpret similar neurodegenerative disorders [16]. Interestingly, CSF α-syn had a strong positive correlation with CSF tau in AD [17–20]. Other studies reported that the levels of α-syn in CSF tended to increase in AD compared to healthy control (HC) [17–19, 21–26]. These results supported that α-syn might be involved in AD pathology through a close relationship with tau.

We examined whether α-syn could contribute in improving the accuracy of AD diagnosis and interpretation of patient groups. For this purpose, the associations of CSF α-syn with other biomarkers in AD, mild cognitive impairment (MCI), subjective cognitive decline (SCD), age-matched HC, and PD as a disease control were investigated.

## Materials and methods

### Participants and sampling

Participants, including early-onset AD (EOAD), late-onset AD (LOAD), MCI, SCD, PD, and HC, were enrolled in Alzheimer’s Disease All Markers study from the multicenter in South Korea. The clinical diagnosis of AD was as following: (1) probable AD criteria proposed by NIA-AA (National Institute on Aging-Alzheimer’s Association workgroups, 2011); (2) male or female patient between the age of 50 and 80, (3) education years of at least 6 years; (4) follow-up at least 6 months to determine the clinical course of AD by experienced neurologists. AD was classified as EOAD and LOAD by a fiducial line at the age of onset of 65 years. Diagnosis of MCI was made according to the presence of impairment in one or more cognitive domains, but the functional abilities are preserved and without meeting the criteria for dementia due to AD set forth by the NIA-AA criteria. The group of SCD was diagnosed according to the presence of subjective cognitive decline at a normal test performance level based on the detailed neuropsychological tests. The criteria for the HC group in the study were the following: (1) a community-based population; (2) no abnormality on the Heath Screening Questionnaire; (3) absence of memory complaints; (4) the Korean Dementia Screening Questionnaire ≤ 6; (5) normal general cognition (within 1 standard deviation of the age- and education-adjusted norms of MMSE and a score higher than 26); (6) intact activities of daily living (K-IADL ≤ 0.42); (7) no depression (the short-form Geriatric Depression Scale ≤ 7); and (8) no history of thyroid dysfunction, vitamin B12 deficiency, or folate deficiency. PD patients were recruited according to the UK Parkinson’s Disease Society Brain Bank Criteria. Exclusion criteria included the cognitive impairment other than AD, stroke, and delirium.

### CSF collection and processing

CSF was obtained from a routine lumbar puncture in the L3/L4 or L4/L5 interspace between 8 to 12 am. The first 4 mL was used for routine analyses including cell count, protein, and sugar levels. To separate the supernatant, CSF was centrifuged at 2000×*g* for 10 min within 4 h from the lumbar puncture. The supernatant was aliquoted into 1 mL of polypropylene vials and stored at − 80 °C until their use. Since large concentrations of α-synuclein existed in red blood cells, the contaminated CSF samples from hemolysis were excluded from the measurement.

### Amyloid-PET acquisition and processing

Amyloid-PET was executed for several individuals except patients with PD. 18F-N-(3-fluoropropyl)-2β-carboxymethoxy-3β-(4-iodophenyl) nortropane (FP-CIT) positron emission tomography (PET) image analysis was performed on PD patients. Patients underwent fluorine 18–labeled (18F) florbetaben PET, and computed tomography images were acquired using a 16-slice helical computed tomography (140 KeV, 80 mA; 3.75-mm section width) for attenuation correction. For 18F-florbetaben PET, a 20-min emission PET scan with dynamic mode (consisting of 4 × 5-min frames) was performed 90 min after injection of approximately 300 MBq of 18F-florbetaben. Three-dimensional PET images were reconstructed in a 128 × 128 × 48 matrix with 2 × 2 × 3.27-mm voxel size using the ordered-subsets expectation maximization algorithm (iteration, 4 and subset, 20). We defined amyloid-PET to be positive when visual assessment of florbetaben PET was scored as 2 or 3 on the brain Aβ plaque load (BAPL) scoring system. BAPL scoring depends on the visual assessment by the nuclear medicine specialist in the trans-axial plane based on regional cortical tracer uptake (RCTU) scoring system of the frontal cortex, lateral temporal cortex, posterior cingulate cortex/precuneus, and parietal cortex. A RCTU score of 1 in each brain region results in a BAPL score of 1, a RCTU score of 2 in any brain region and no score 3 result in a BAPL score of 2. A RCTU score of 3 in any of the 4 brain regions results in a BAPL of 3.

### CSF analysis

The levels of CSF Aβ_42_, T-tau, and P-tau_181_ (Triple marker) were measured by commercial ELISA kits (INNOTEST β-AMYLOID(1–42), INNOTEST hTAU-Ag, and INNOTEST PHOSPHO-TAU(181P), Fujirebio Europe, Gent, Belgium) according to the manufacturer’s instructions.

### Quantification of α-synuclein in CSF

An in-house ELISA assay was developed to measure total α-syn in CSF samples. A 96-well Polysorp NUNC microplate (Thermo Fischer Scientific, USA) was coated with the capture antibody (4B12, BioLegend) in coating buffer overnight at 4 °C. The plate was then washed three times with phosphate-buffered saline with 0.05% Tween-20 (PBST) and incubated with 300 μL/well of blocking buffer for 1 h at 37 °C. After three washes with PBST, serially diluted recombinant α-syn and thawed CSF (diluted 3:1 with sample dilution buffer) were applied to each well and incubated for 2.5 h at room temperature (RT). Subsequently, the plate was washed five times and incubated with the biotinylated detection antibody (4D6, BioLegend) in the reaction solution for 1 h at RT. After washing, the plate was incubated with streptavidin poly-HRP (Thermo Fischer Scientific, USA) in the reaction solution for 0.5 h at 37 °C. Finally, the plate was washed five times and reacted with TMB substrate (Thermo Fischer Scientific, USA) for 0.5 h at RT in the dark. The optical density was measured after adding the stop solution. Reagents (AGMIG-0100, Arista biologicals) to mitigate heterophilic antibody interference was included in all assays to remove false signals.

### Statistical analysis

Statistical analyses were performed by SPSS 23 software (SPSS Inc., Chicago, IL). Mann-Whitney *U* test and Kruskal-Wallis test were used to compare demographics and clinical and biomarker values. Chi-squared test and Fisher’s exact test were performed to assess the statistical difference in sex, CDR, and visual amyloid-PET reading among groups. *P* < 0.05 was considered statistically significant. Spearman’s correlation was used to examine the relationship between different biomarkers. The area under the curve (AUC) in the receiver operator characteristic (ROC) curve was analyzed to evaluate the accuracy of the diagnostic value of biomarkers. Cutoff values were obtained according to the sensitivity and specificity at the point where the Youden index is maximized. Percentage agreement was used to quantify concordance between biomarkers. Overall percentage agreement (OPA) reflects the percentage of diagnostic matches between the two biomarkers. Positive percent agreement (PPA) and negative percent agreement (NPA) were defined as sensitivity (a percentage that both biomarkers diagnose as AD) and specificity (a percentage that both biomarkers diagnose as HC), respectively. The clustering of samples based on biomarker profiles was analyzed using an analysis of similarities (ANOSIM) to calculate statistically difference between groups.

## Results

### Demographics and biomarker values

Demographic characteristics, clinical features, and CSF biomarker levels of all participants were reported in Table 1. EOAD and LOAD revealed typical AD levels in triple biomarkers, and especially EOAD tended to progress more than LOAD without statistical difference. The concentrations of biomarkers in MCI and SCD were distributed near the intermediate region between AD and HC. In the case of PD, the reduced levels of Aβ_42_ were observed as in MCI, and both T-tau and P-tau_181_ were statistically lower than in HC. The concentrations of α-syn had no statistical significance between PD and HC. However, the increasing tendency of α-syn in EOAD and LOAD was noticeable, whereas the reduced α-syn was seen in PD than HC. Hence, the reduced levels of α-syn in PD had statistical significance in comparison with EOAD, LOAD, MCI, and SCD. When the AD group with positive triple markers and the HC group with negative triple markers were selected, the levels of α-syn were significantly different between the groups (Additional file 1: Fig. S1a). The concentrations of α-syn were slightly higher in AD with positive amyloid-PET than HC with negative amyloid-PET without statistical significance (Additional file 1: Fig. S1b). Additionally, α-syn was highly elevated in APOEε4 homozygotes carrier group (Additional file 1: Fig. S1c).

**Table 1:** Demographics and biomarker values for the diagnostic groups^a^

	EOAD	LOAD	MCI	SCD	PD	HC	*P*
*N*	71	34	11	17	45	32	
Sex (M/F)	25/46	17/17	6/5	4/13	18/27	12/20	.412^b^
Age^c^	58.0 (54.0–62.0)	77.0 (73.0–80.0)	68.0 (59.0–75.0)	67.0 (61.0–73.0)	68.0 (60.0–74.5)	66.5 (62.0–68.0)	.000^d^
MMSE^e^	16.0 (13.0–20.0)	19.0 (16.0–21.0)	25.0 (24.0–26.0)	29.0 (25.5–29.0)	–	28.0 (27.0–30.0)	.000^d^
CDR 0/0.5/1/2/3^e^	0/20/33/15/3	0/13/15/6/0	0/10/1/0/0	2/15/0/0/0	–	15/17/0/0/0	.000^f^
CSF Aβ_42_ (pg/mL)	326.8 (258.6–488.2)	401.3 (239.6–587.3)	737.5 (445.9–806.9)	990.3 (904.3–1043.5)	774.4 (595.2–967.2)	1078.0 (982.4–1297.3)	.000^d^
CSF T-tau (pg/mL)	534.3 (386.2–732.9)	442.6 (326.3–598.2)	283.6 (203.7–490.8)	321.9 (252.1–335.9)	159.4 (116.9–215.8)	203.3 (147.1–295.9)	.000^d^
CSF P-tau_181_ (pg/mL)	77.2 (53.9–106.0)	69.9 (44.1–89.7)	54.6 (40.1–83.3)	51.5 (41.1–60.4)	34.8 (25.5–43.6)	40.3 (32.1–54.6)	.000^d^
CSF α-syn (pg/mL)	754.2 (526.7–1163.9)	834.8 (586.5–1330.3)	889.5 (628.7–1079.5)	880.4 (689.5–1154.8)	590.9 (420.0–842.3)	669.8 (506.8–973.3)	.007^d^
Amyloid-PET (+/−)^g^	50/1	18/10	1/4	1/12	–	1/21	.000^b^

*Abbreviations*: *EOAD* early-onset Alzheimer’s disease, *LOAD* late-onset Alzheimer’s disease, *MCI* mild cognitive impairment, *SCD* subjective cognitive decline, *PD* Parkinson’s disease, *HC* healthy control, *M* male, *F* female, *MMSE* Mini-Mental State Examination, *CDR* Clinical Dementia Rating, *CSF* cerebrospinal fluid, *Aβ*_*42*_ amyloid-β 1-42; *T-tau* total tau; *P-tau*_*181*_ phosphorylated tau 181, *α-syn* α-synuclein, *FBB-PET*^18^F-florbetaben positron emission tomography

^a^Data are shown as median (interquartile) unless otherwise indicated

^b^Chi-square test

^c^Excluding EOAD and LOAD, the *P* value is 0.425

^d^Kruskal-Wallis test

^e^PD was not performed the cognitive assessment

^f^Fisher’s exact test

^g^A limited number of individuals were available for amyloid-PET scan

### Pendulum phenomenon at the balance of tau and α-syn

**Fig. 1:**
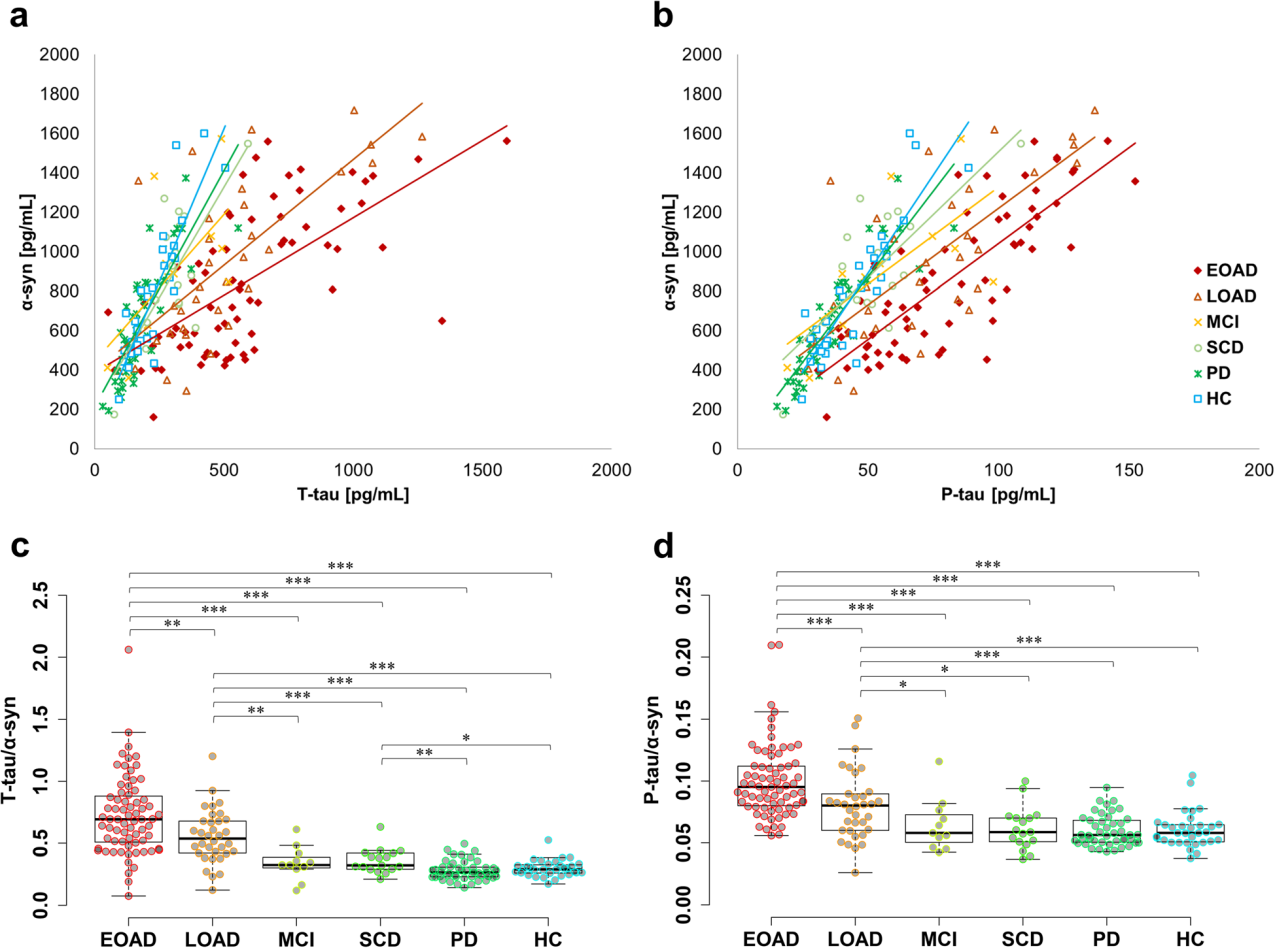
The balance between α-syn and tau in each group. **a**, **b** Scatter plot of α-syn and tau values. Each group was shown in different colors and shapes, and the slope was indicated with the same color as each group. **c**, **d** The value of T-tau/α-syn and P-tau_181_/α-syn were indicated by dot plots. Box plots display median, first and third quartile. *P < 0.05, **P < 0.01, and ***P < 0.001

**Fig. 2:**
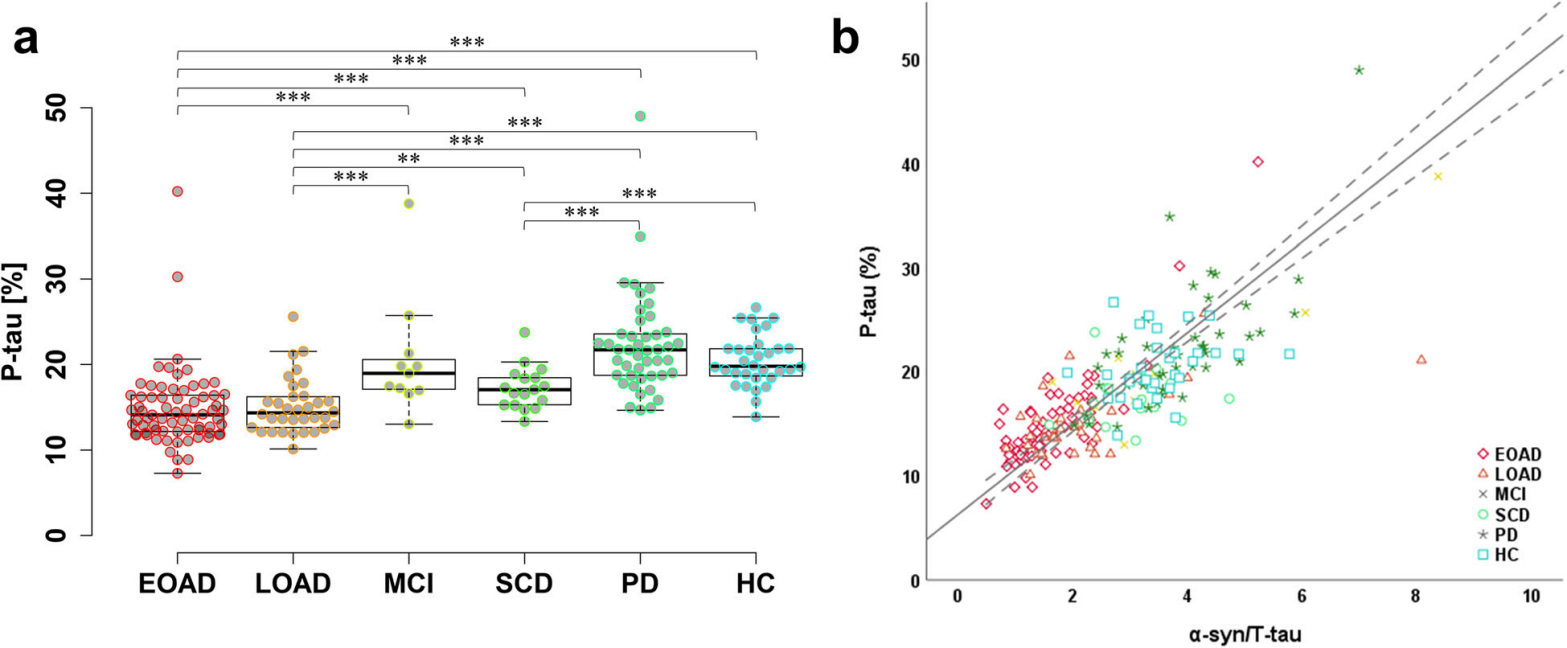
The percentage of tau phosphorylation and its correlation with the ratio of T-tau and α-syn. **a** The percentage of phosphorylated tau in each group. **b** The correlation of phosphorylated tau percentage with the ratio of α-syn to T-tau concentrations. *P < 0.05, **P < 0.01, and ***P < 0.001

α-Syn had significant positive correlations with T-tau and P-tau_181_ with correlation coefficients at 0.619 and 0.737_,_ respectively. Remarkably, when the correlation graph was divided into each group, the gradient decreased gradually from HC to AD (Fig. 1a, b). The ratios of individual T-tau/α-syn and P-tau_181_/α-syn were calculated and plotted as dot and box plots for comparing gradient differences in each group (Fig. 1c, d). As a result, T-tau/α-syn and P-tau_181_/α-syn were significantly higher in AD than in MCI, SCD, PD, or HC. For T-tau/α-syn, the differences between AD and HC were greater than P-tau_181_/α-syn, and the values of AD and HC groups were highly segregated. SCD also had a significant difference with PD and HC in T-tau/α-syn. MCI showed a similar tendency as SCD, but there was no statistical significance possibly due to the insufficient number of samples. Interestingly, T-tau/α-syn and P-tau_181_/α-syn appeared the statistical difference between EOAD and LOAD. On the other hand, although P-tau_181_ was increased in AD, the percentage of P-tau_181_ was significantly decreased in AD compared to HC (Fig. 2a). Surprisingly, α-syn/T-tau, which was inverted from T-tau/α-syn for a correlation analysis, had a strong correlation with tau phosphorylation rate in Fig. 2b (correlation coefficient = 0.821; *P* < 0.001).

### ROC curve analysis

ROC curve analysis was conducted to investigate the contributions of α-syn in improving diagnostic accuracy (Table 2). EOAD and LOAD were categorized into AD group, PD and HC were set in non-AD group. In the case of Aβ_42_, cutoff and AUC were lowered to 555.4 pg/mL and 0.899, respectively, since PD group had low concentrations of Aβ_42_. The AUC of both T-tau and P-tau_181_ were 0.908 and 0.860, respectively. In particular, the incorporation of α-syn improved AUC value of T-tau/α-syn to 0.930. Moreover, AUC was the highest at 0.956 (specificity = 96%, sensitivity = 87%) from the composite biomarkers (Aβ_42_/P-tau_181_ and T-tau/α-syn) without statistical significance in comparison to Aβ_42_/P-tau_181_ and T-tau/α-syn (Additional file 1: Supplement Table 2).

**Table 2:** Analysis of ROC curve for each biomarker between EOAD, LOAD versus PD, and HC

CSF biomarker	Cutoff	AUC	95% CI	Sensitivity (%)	Specificity (%)
Aβ_42_	555.4^a^	0.899	0.846–0.939	90	81
T-tau	318.9^a^	0.908	0.857–0.946	81	91
P-tau_181_	57.6^a^	0.860	0.801–0.907	67	90
Aβ_42_/P-tau_181_	10.6	0.948	0.905–0.976	92	90
T-tau/α-syn	0.412	0.930	0.883–0.962	89	95
P-tau_181_/α-syn	0.071	0.869	0.811–0.914	80	82
Aβ_42_/P-tau_181_, T-tau/α-syn	21.5	0.956	0.915–0.981	96	87

*Abbreviation*: *ROC* receiver operator characteristic, *EOAD* early-onset Alzheimer’s disease, *LOAD* late-onset Alzheimer’s disease, *PD* Parkinson’s disease, *HC* healthy control, *CSF* cerebrospinal fluid, *AUC* area under the curve, *Aβ*_*42*_ amyloid-β 1-42, *T-tau* total tau, *P-tau*_*181*_ phosphorylated tau 181, *α-syn* α-synuclein

^a^Unit: pg/mL

### Concordance between CSF biomarkers

**Fig. 3:**
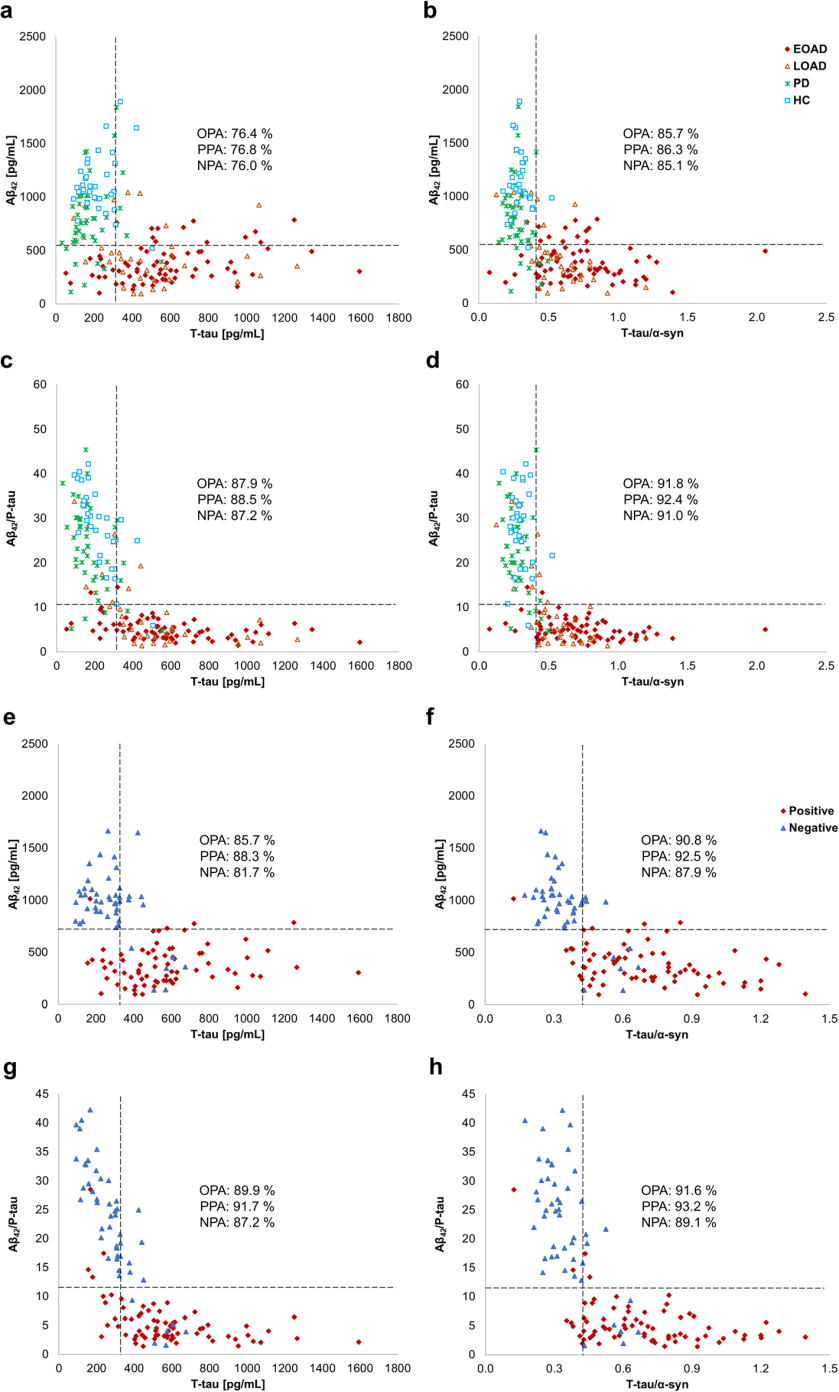
Concordance between biomarkers in groups defined by clinical or amyloid-PET. **a**–**d** groups classified by clinical. **e**–**h** groups classified by amyloid-PET. Scatter plot: **a**, **e** Aβ_42_ versus T-tau, **b**, **f** Aβ_42_ versus T-tau/α-syn, **c**, **g** Aβ_42_/P-tau_181_ versus T-tau, and **d**, **h** Aβ_42_/P-tau_181_ versus T-tau/α-syn. Vertical and horizontal dashed lines indicate the cutoff of each biomarker

Using pre-defined cutoffs, OPA, PPA, and NPA were calculated to determine the degree of concordance between CSF biomarkers, and the level of each biomarker was displayed as a scatter plot (Fig. 3a–d). The concordances of Aβ_42_ with T-tau were 76, 77, and 76% for OPA, PPA, and NPA, respectively. On the other hand, T-tau/α-syn showed better agreement with Aβ_42_ than T-tau (OPA = 86%, PPA = 86%, NPA = 85%). When Aβ_42_/P-tau_181_ and T-tau/α-syn were compared, the concordance rate of diagnosis had the highest values (OPA = 92%, PPA = 92%, NPA = 91%). Next, the concordance of the biomarkers in amyloid-PET positive and negative groups was examined (Fig. 3e–h). Interreader agreements between Aβ_42_ and T-tau were 86, 88, and 82% for OPA, PPA, and NPA, respectively. Otherwise, T-tau/α-syn had higher OPA, PPA, and NPA with Aβ_42_ than T-tau (OPA = 91%, PPA = 93%, NPA = 88%). Especially, Aβ_42_/P-tau_181_ and T-tau/α-syn almost similarly distinguished the amyloid-PET classification (OPA = 92%, PPA = 93%, NPA = 89%).

### Quadruple biomarker profiling

The distributions of individuals classified as ATN (A = Aβ_42_, T = P-tau_181_, and N = T-tau) were examined in the clinically diagnosed group (Table 3). T-tau/α-syn was marked as N/αS, and pre-defined cutoffs were used to divide the biomarkers into positive (+) and negative (−). Additionally, the percentage of N/αS+ was calculated for each group. In groups based on the clinical judgment, the percentage of N/αS+ was the highest with 90% in EOAD, followed by 77% in LOAD. The percentage of N/αS+ was 18% in MCI similar to 24% in SCD. PD and HC revealed the lowest N/αS+ percentage of 7% and 3%, respectively. On the other hand, when the ratio of N/αS+ was analyzed in the classified group as ATN, it was higher in the A+T−N+ (100%) and the A+T+N+ group (92%). The percentage of N/αS+ was 59% in A+T−N−, which was lower at 5% in A−T−N−. Interestingly, among groups in the suspected non-Alzheimer’s pathology physiology (SNAP), N/αS+ percentages were 40% in A−T+N+, and both A−T-N+ and A−T+N− were 0%.

**Table 3:** Categorization according to clinical diagnosis and ATN biomarker profiles

ATN profiles			EOAD	LOAD	MCI	SCD	PD	HC	N/αS+ (%)
	A−T−N−	N/αS−	1	4	5	8	34	27	5
		N/αS+	–	–	–	3	–	1	
	A+T−N−	N/αS−	3	1	1	–	4	–	59
		N/αS+	8	3	–	–	2	–	
AD	A+T+N−	N/αS−	1	–	–	–	–	–	0
		N/αS+	–	–	–	–	–	–	
	A+T+N+	N/αS−	2	1	–	1	–	1	92
		N/αS+	41	17	2	–	1	–	
	A+T−N+	N/αS−	–	–	–	–	–	–	100
		N/αS+	10	5	–	1	–	–	
SNAP	A−T−N+	N/αS−	–	1	–	1	1	–	0
		N/αS+	–	–	–	–	–	–	
	A−T+N−	N/αS−	–	–	1	1	1	1	0
		N/αS+	–	–	–	–	–	–	
	A−T+N+	N/αS-	–	1	2	2	2	2	40
		N/αS+	5	1	–	–	–	–	
N/αS+ (%)			90	77	18	24	7	3	

*Abbreviations*: *AD* Alzheimer’s disease, *EOAD* early-onset Alzheimer’s disease, *LOAD* late-onset Alzheimer’s disease, *MCI* mild cognitive impairment, *SCD* subjective cognitive decline, *PD* Parkinson’s disease, *HC* healthy control, *SNAP* suspected non-Alzheimer’s disease pathophysiology

### α-Syn as a biomarker of differential diagnosis in neurodegeneration

**Fig. 4:**
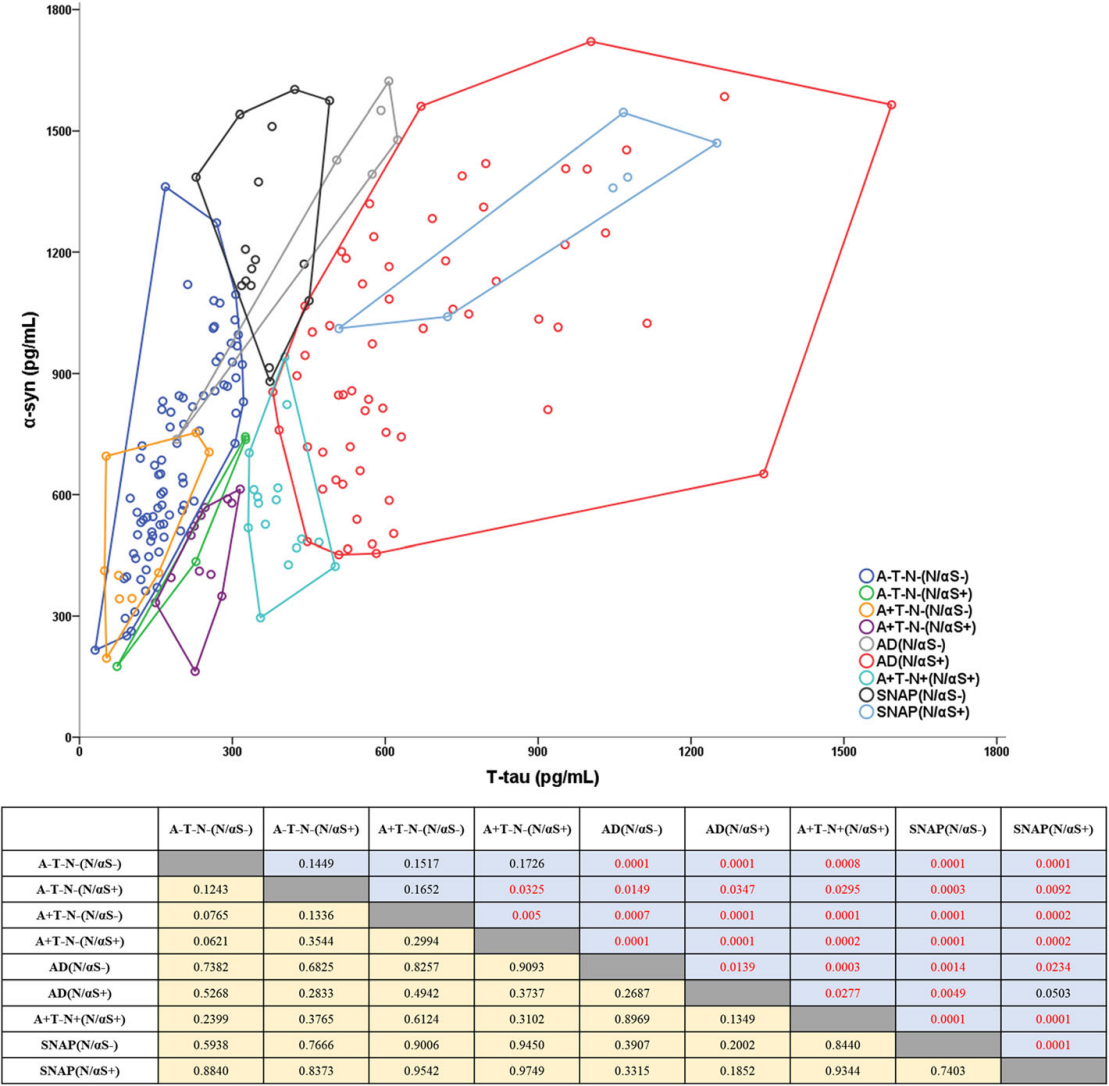
Distribution of T-tau and α-syn in ATN(N/αS) groups. The clustering of samples was visualized in a scatter plot with Convex hulls. The table shows the difference between the two groups as *P* value (blue) and *R* value (yellow). Statistically significant values were colored in red

Remarkably, ATN(N/αS) groups showed different tendencies when T-tau and α-syn levels were visualized in the scatter plot (Fig. 4). Most groups were clustered and distributed without overlapping. Statistically, *P* value and *R* value were calculated by ANOSIM to quantify the differences between the clusters. *P* value of less than 0.05 was considered statistically significant, and *R* value closer to one meant that the difference between the two clusters would have a higher significance. AD group included A+T+N− and A+T+N+, and SNAP consisted with A−T−N+, A−T+N−, and A−T+N+. A+T−N−(N/αS−) belonged to A−T−N−(N/αS−), and A−T−N−(N/αS+) and A+T−N−(N/αS+) clustered to similar positions. Interestingly, SNAP(N/αS−) revealed to be an extended position in A−T−N−(N/αS−) and was markedly distinguished from AD. Moreover, SNAP(N/αS+) was included in the cluster of AD(N/αS+) and was recognized as the same group. Although the number of samples was limited, AD(N/αS−) and A+T−N+(N/αS+) were distributed independently from other groups.

## Discussion

Recently, α-syn has been considered to play an important role in AD. Previous studies reported that CSF α-syn had a high positive correlation with tau and tended to increase in AD compared to HC [17–24, 27, 28]. Our results corroborated the previous finding that α-syn had a tendency to increase in AD (Table 1), which was significantly correlated with T-tau and P-tau_181_ in CSF (Fig. 1a, b). Especially, the levels of α-syn were elevated in AD patients with positive CSF triple markers and APOEε4 homozygous carriers (Additional file 1: Fig. S1). The increases of CSF α-syn in AD were not enough to be used as a biomarker, but these results strongly suggested the potential role of α-syn in AD pathophysiology.

Interestingly, the equilibrium states of tau and α-syn concentrations were changed in AD, and the ratios of T-tau/α-syn and P-tau_181_/α-syn were elevated with the progression from HC to AD (Fig. 1a–d). A review paper by Moussaud et al. suggested a putative pathway of α-syn for the tau neurofibrillary tangles [29]. α-Syn could interact with the microtubule-binding region of tau through its C-terminus, inhibiting the binding of tau to tubulin and causing the increased concentrations of free tau [30]. α-Syn also could bind to tubulin directly and may promote tubulin polymerization [31, 32]. These findings implicated that α-syn might be directly involved in tauopathy. On the other hand, α-syn accelerated tau phosphorylation through direct binding with GSK-3β and tau from in vitro study [33]. Tau phosphorylation by GSK-3β was elevated as α-syn/T-tau ratio increased [33]. Based on these results, increased α-syn in AD may induce tau phosphorylation with GSK-3β. However, the percentage of P-tau_181_ was decreased in AD (Fig. 2a), despite increasing absolute concentrations of P-tau_181_ in AD. Remarkably, the rate of P-tau_181_ had a strong positive correlation with α-syn/T-tau ratio in all groups (Fig. 2b; correlation coefficient, 0.821), and the compatible results were obtained with the direct correlation between tau phosphorylation and α-syn/T-tau ratio, similar the previous reports. Even though the levels of α-syn increased slightly in AD, the percentage of P-tau_181_ was the lowest in AD, coinciding with the drastic decreased α-syn/T-tau ratio. It did not prove whether the P-tau_181_ phosphorylation rate was determined by the ratio of α-syn and tau concentrations, but the results suggested that the ratio of T-tau and α-syn could be a reliable indicator for the phosphorylation of tau regardless of the type of disease.

Interestingly, the ratios of T-tau/α-syn and P-tau_181_/α-syn were statistically different in EOAD and LOAD in Fig. 1c, d (*P* = 0.004 and *P* = 0.001, respectively). The previous study reported that autosomal dominant AD (ADAD) mutation carriers had lower Aβ_42_ and higher tau concentrations in CSF than non-carriers in the expected period of symptom onset [34]. Moreover, CSF α-syn levels were higher in symptomatic ADAD mutation carriers than in non-mutation carriers [28]. Although the precise mechanism was not clear, these results implicated that α-syn was highly involved in ADAD with genetic factors, resulting in significant differences in T-tau/α-syn and P-tau_181_/α-syn between EOAD and LOAD. In addition, α-syn was significantly elevated in the APOEε4 homozygous carrier (Additional file 1: Fig. S1c). The previous study also revealed a positive correlation with α-syn and APOEε4 alleles, and suggested the promotion of α-syn-derived pathology by APOE [28]. The increased α-syn levels in EOAD and APOEε4 homozygous carrier suggested the importance of α-syn in AD pathophysiology and further investigations about the association between α-syn and AD-related genes.

Previously, α-syn was reported with other biomarkers for improving the accuracy of neurodegenerative disorders diagnosis [17, 35]. In the current study, the incorporation of α-syn confirmed the better performance in the ROC curve analysis (Table 2). The sensitivity and specificity of T-tau/α-syn were 89% and 95%, respectively. Especially, the quadruple biomarker (Aβ_42_/P-tau_181_ and T-tau/α-syn) showed the best results (AUC = 0.956, sensitivity = 96%, specificity = 87%). Also, T-tau/α-syn improved concordance analysis by reducing the disagreement of T-tau versus Aβ_42_ and amyloid-PET (Fig. 3) and enhanced the correlations with MMSE and CDR (Additional file 1: Fig. S2). In the dynamic change model of biomarkers in AD, CSF Aβ_42_ preferentially preceded before disease outset, followed by amyloid-PET and CSF tau, sequentially [36]. Interpreting our results based on this model, the results could be inferred that the changes of T-tau/α-syn might be a time point similar to the change in Aβ_42_. In addition, T-tau/α-syn had a statistical increase in SCD over HC (Fig. 1c), indicating its potential as a prognostic biomarker. In previous studies, α-syn showed a positive association with brain amyloid beta deposition in the cognitively normal subject with memory complain and was the highest level in patients with MCI, suggesting the involvement of α-syn from the early stage of AD [20, 35]. In view of all findings, α-syn might play a role with the association of amyloid beta deposition from the preclinical stage of AD and could improve prognostic and diagnostic performance as a biomarker in AD.

In 2018, NIA-AA published a research framework for observational and interventional research [16]. They recommended a guideline for diagnosing AD by dividing the biomarker group into three groups: amyloid beta deposition (A), pathological tau (T), and neurodegeneration (N). In this study, clinically well-characterized groups revealed typical ATN profiling results and were additionally classified by N/αS, meaning T-tau/α-syn (Table 3). N/αS+ group percentage was the highest in EOAD and LOAD, and the percentage decreased gradually in MCI, SCD, PD, and HC, suggesting that N/αS specifically reflected the underlying pathology of AD. Based on Fig. 4 and Table 3, the possible meaning of each ATN(N/αS) group was examined to verify that N/αS group classification helped the diagnosis, as inferred in Supplementary Table 1. Interestingly, all groups had distinctive characteristics, which supported the importance of N/αS incorporation in the AD panel. Remarkably, SNAP(N/αS+) included only AD patients with A−T+N+ and clustered completely belonged to AD(N/αS+) group. In other studies, the group classified as A−T+N+ due to higher concentrations of Aβ_42_, despite having amyloid-PET positive, was reintegrated into the AD spectrum by applying Aβ_42/40_ ratio [37, 38]. Based on previous reports, SNAP(N/αS+) could be misclassified into SNAP possibly due to the over-production of Aβ_42_. In SNAP(N/αS-), T-tau and α-syn were located on the extension of A−T−N−(N/αS−) without a change in the equilibrium state and clustered separately from AD. This group may have its own pathophysiology such as frontotemporal dementia and may be associated with over-production of α-syn.

In the current study, the inconsideration of oligomeric α-syn in CSF could be the limitations. Synucleinopathies related to oligomeric α-syn might alter the total α-syn levels in AD. Another limitation could be the associative proteins with α-syn were not included in the study, especially when α-syn was directly involved in the process of vesicle trafficking. In addition, other proteins, such as LRRK or DJ-1, in association with α-syn should be considered, which would support the involvement of α-syn in AD.

## Conclusion

The findings of α-syn classification may provide better accurate distinctions between AD and Alzheimer’s pathological changes, especially between EOAD and LOAD and from SNAP. Subsequently, if clear pathological pathways of constituting N/αS would be developed, the ATN criteria could be extended by accepting new biomarkers, in composing ATN(N/αS) classification. Although α-syn was not sufficient as a stand-alone biomarker, the incorporation of α-syn is expected to serve as a useful biomarker for explaining the cumulative and precise diseases of neurological damages sensitively and specifically in the future.

## Supplementary information

**Additional file 1.**

## Data Availability

The datasets used and/or analyzed in the current study are available from the corresponding author on reasonable request.

## Abbreviations

Aβ_42_: Amyloid-β1-42
AUC: Area under the curve
BAPL: Brain Aβ plaque load
EOAD: Early-onset Alzheimer’s disease
FP-CIT: 18F-N-(3-fluoropropyl)-2β-carboxymethoxy-3β-(4-iodophenyl) nortropane
HC: Healthy control
LOAD: Late-onset Alzheimer’s disease
MCI: Mild cognitive impairment
NPA: Negative percent agreement
OPA: Overall percentage agreement
PD: Parkinson’s disease
PPA: Positive percent agreement
PBST: Phosphate-buffered saline with 0.05% Tween-20
P-tau_181_: Phosphorylated tau181
RCTU: Regional cortical tracer uptake
RT: Room temperature
ROC: Receiver operator characteristic
SCD: Subjective cognitive decline
SNAP: Suspected non-Alzheimer’s pathology physiology
T-tau: Total tau
18F: Fluorine 18–labeled
α-syn: α-synuclein
