# Supplementary material for: CSF total tau/α-synuclein ratio improved the diagnostic performance for Alzheimer’s disease as an indicator of tau phosphorylation
Source: Alzheimers Res Ther. 2020 Jul 13;12:83. doi: 10.1186/s13195-020-00648-9 (PMC7359621; doi:10.1186/s13195-020-00648-9)
Supplement: Supplementary file 1 — Additional file 1. [file 13195_2020_648_MOESM1_ESM.docx]

**Supplement Contents**


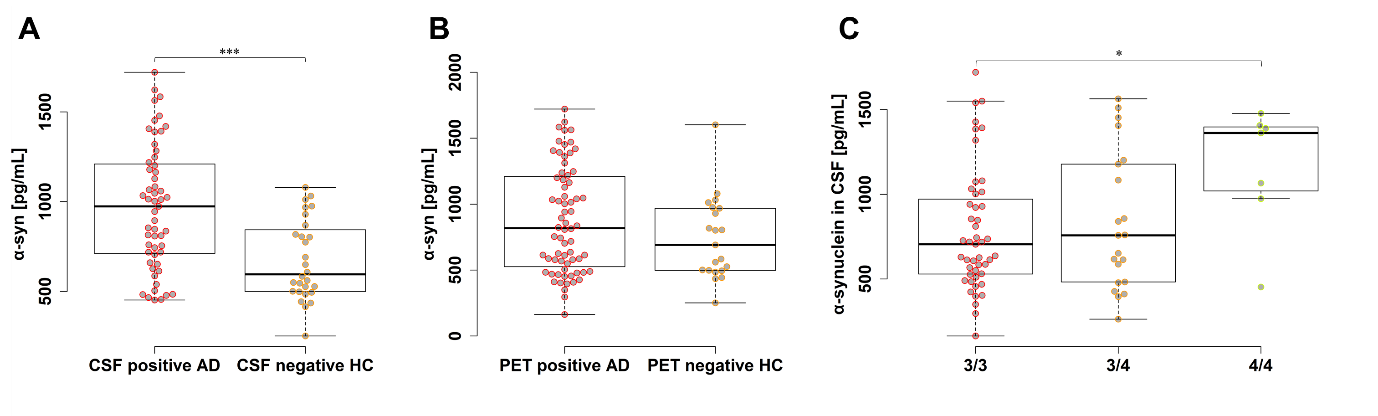


Supplement Figure 1 The levels of α-syn between two groups selected by CSF biomarker value, the visual PET reading, and APOEε genotype. (A) AD patients with all positive CSF triple marker levels were sorted as CSF-positive. Oppositely, the CSF-negative group involves HC with all negative CSF triple marker levels. (B) PET-positive and -negative groups were classified through visual PET reading from AD and HC, respectively. (C) groups were divided by APOEε genotype. Abbreviation: α-syn, α-synuclein; CSF, cerebrospinal fluid; PET, positron emission tomography; AD, Alzheimer’s disease; HC, healthy control.


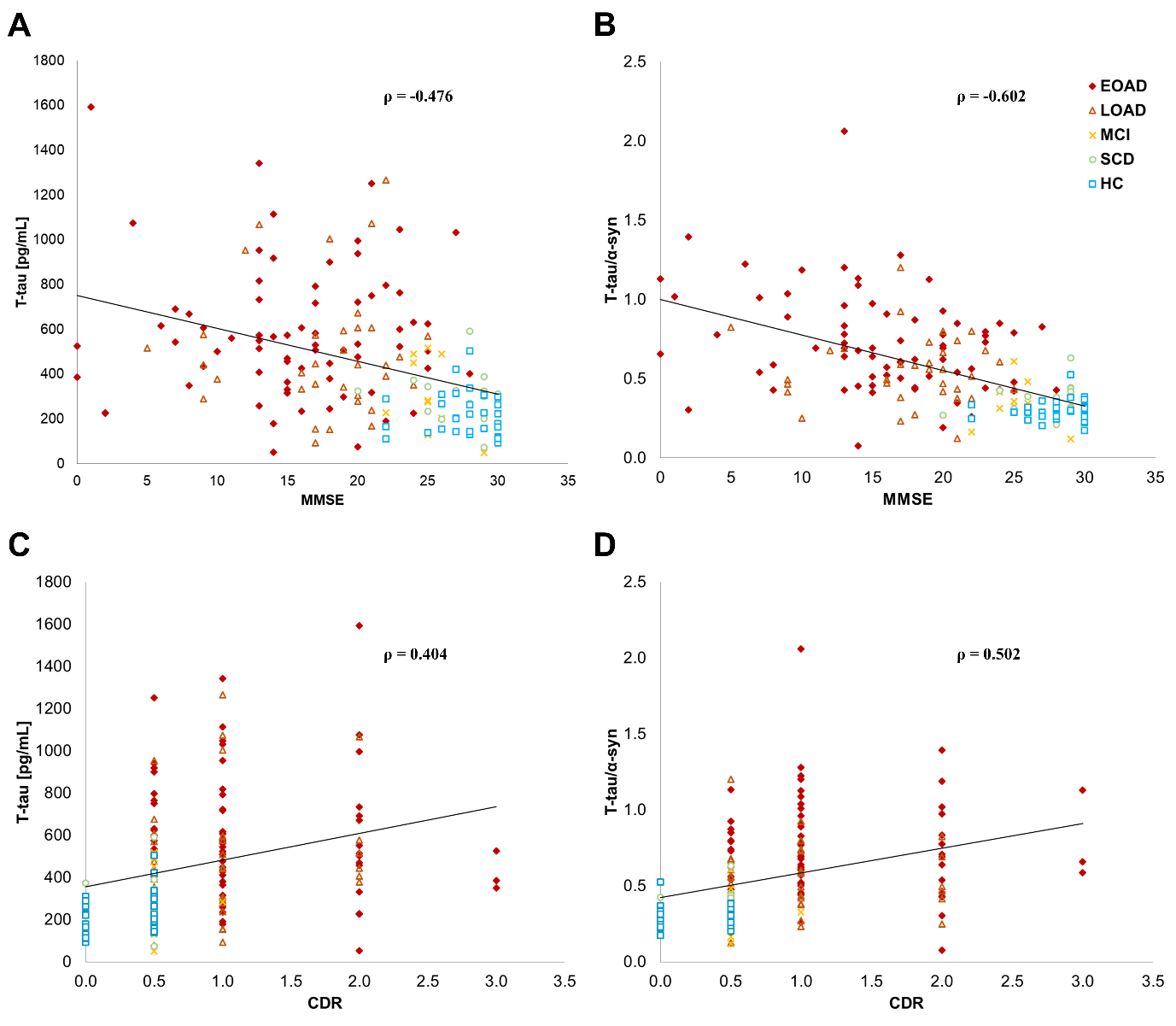


Supplement Figure 2 Correlation with cognitive decline and biomarkers. (A) T-tau and MMSE. (B) T-tau/α-syn ratio and MMSE. (C) T-tau and CDR. (D) T-tau/α-syn ratio and CDR. Abbreviation: MMSE, Mini Mental State Examination; CDR, Clinical Dementia Rating; EOAD, early-onset Alzheimer’s disease; LOAD, late-onset Alzheimer’s disease; MCI, mild cognitive impairment; SCD, subjective cognitive decline; PD, Parkinson’s disease; HC, healthy control; T-tau, total tau; P-tau_181_, phosphorylated tau 181; α-syn, α-synuclein.


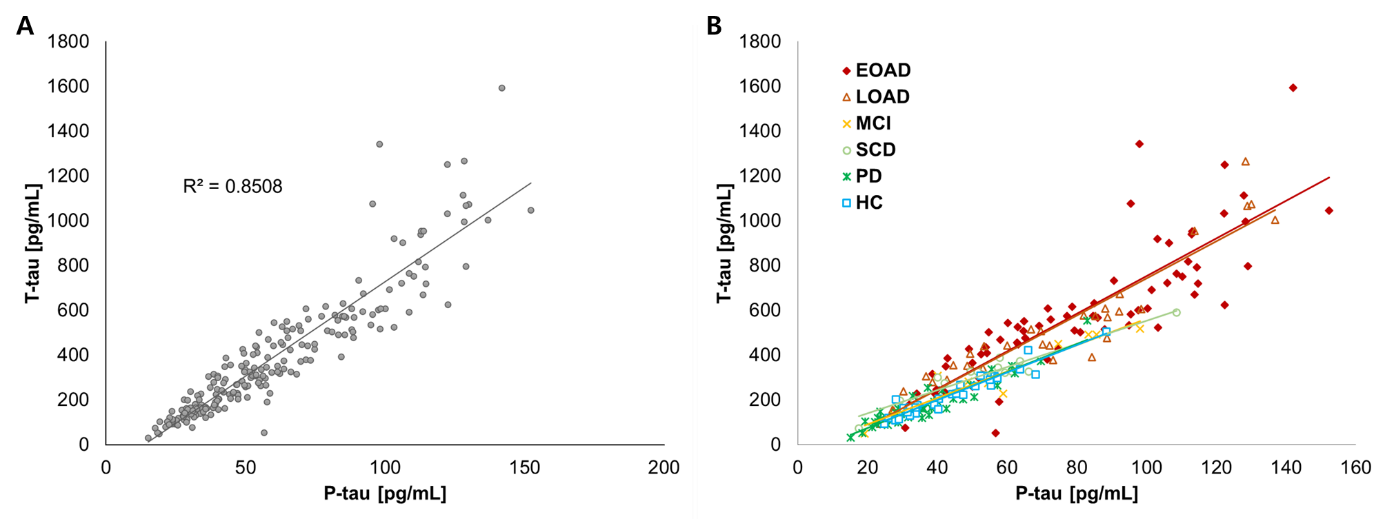


Supplement Figure 3 Correlation between T-tau and P-tau_181_. (A) T-tau and P-tau_181_. (B) T-tau and P-tau_181_ in each group.

Abbreviation: EOAD, early-onset Alzheimer’s disease; LOAD, late-onset Alzheimer’s disease; MCI, mild cognitive impairment; SCD, subjective cognitive decline; PD, Parkinson’s disease; HC, healthy control; T-tau, total tau; P-tau_181_, phosphorylated tau 181.

**Supplement Table 1** Quadruple CSF biomarkers profiles and characteristics

| **ATN profiles** | | **Characteristics** |
| --- | --- | --- |
| A-T-N- | N/αS- | Normal AD biomarkers |
|  | N/αS+ | Possible neurodegenerative change with tau and α-synuclein pathophysiology |
| A+T-N- | N/αS- | Alzheimer’s pathologic change or concomitant with synucleinopathies |
|  | N/αS+ | Alzheimer’s pathologic change with tau and α-synuclein pathophysiology |
| AD | N/αS- | AD with over-production/secretion of α-synuclein or concomitant with other neurodegenerative disorders |
|  | N/αS+ | AD with tau and α-synuclein pathophysiology |
| A+T-N+ | N/αS- | - |
|  | N/αS+ | Alzheimer’s and concomitant suspected non-Alzheimer’s pathologic change with tau and α-synuclein pathophysiology |
| SNAP | N/αS- | Non-Alzheimer’s pathologic change with over-production/secretion of α-synuclein |
|  | N/αS+ | Possibly preclinical AD with over-production/secretion of Aβ_42_ |

Abbreviations: AD, Alzheimer’s disease; Pre-AD, preclinical Alzheimer’s disease; SNAP, suspected non-Alzheimer’s disease pathophysiology.

**Supplement Table 2** Quadruple CSF biomarkers profiles and characteristics

|  | **T-tau** | **P-tau_181_** | **Aβ_42_/**  **P-tau_181_** | **T-tau/**  **α-syn** | **P-tau_181_/**  **α-syn** | **Combined** |
| --- | --- | --- | --- | --- | --- | --- |
| **Aβ_42_** | 0.775 | 0.273 | 0.009 | 0.261 | 0.031 | 0.009 |
| **T-tau** |  | 0.002 | 0.035 | 0.197 | 0.215 | 0.006 |
| **P-tau_181_** |  |  | <0.001 | 0.011 | 0.791 | <0.001 |
| **Aβ_42_/**  **P-tau_181_** |  |  |  | 0.336 | 0.001 | 0.318 |
| **T-tau/**  **α-syn** |  |  |  |  | 0.014 | 0.069 |
| **P-tau_181_/**  **α-syn** |  |  |  |  |  | <0.001 |

^*^The values with *P* < 0.05 are underlined.

^*^Combined means ‘Aβ_42_/P-tau_181_, T-tau/α-syn’
